# Modeling of Biological Intelligence for SCM System Optimization

**DOI:** 10.1155/2012/769702

**Published:** 2011-11-24

**Authors:** Shengyong Chen, Yujun Zheng, Carlo Cattani, Wanliang Wang

**Affiliations:** ^1^College of Computer Science & Technology, Zhejiang University of Technology, Hangzhou 310023, China; ^2^Department of Mathematics, University of Salerno, Via Ponte Don Melillo, 84084 Fisciano, Italy

## Abstract

This article summarizes some methods from biological intelligence for modeling and optimization of supply chain management (SCM) systems, including genetic algorithms, evolutionary programming, differential evolution, swarm intelligence, artificial immune, and other biological intelligence related methods. An SCM system is adaptive, dynamic, open self-organizing, which is maintained by flows of information, materials, goods, funds, and energy. Traditional methods for modeling and optimizing complex SCM systems require huge amounts of computing resources, and biological intelligence-based solutions can often provide valuable alternatives for efficiently solving problems. The paper summarizes the recent related methods for the design and optimization of SCM systems, which covers the most widely used genetic algorithms and other evolutionary algorithms.

## 1. Introduction

Supply chains are a kind of network with facilities and distribution entities (suppliers, manufacturers, distributors, retailers). The supply chain performs the functions of procurement of raw materials, transformation of raw materials into intermediate and finished products, and distribution of finished products to customers [1]. Due to rising product and market complexity, expanding competition, shorter product lifecycles, and changing customer demands, today's supply chain management (SCM) systems increasingly involve complex sets of objectives and constraints, and variations of uncertainty and randomization [2], and thus static and/or centralized models are insufficient to effectively plan, coordinate, and optimize activities in a supply chain.

Modeling and optimization of an SCM system provide a critical support for decision making in a competitive market. According to [3], the basic approaches to supply chain modeling and optimization can be divided into five classes:

1. fundamental formulation of supply chains,
2. integer-mixed programming optimization,
3. stochastic programming,
4. heuristic methods,
5. simulation-based methods.

Traditional simulation methods for large-scale complex systems require huge amounts of computing resources [4]. In recent years, bioinspired methods have gained increasing interest in the research of modeling and optimization for SCM systems which are typically dynamic, open self-organizing systems maintained by flows of information, materials, goods, funds, and energy. Bioinspired and living system mechanisms, such as learning, growth, evolution, collaboration, and competition, bring an innovative solution for the analysis and improvement of emergent complex behaviors in virtually computing modules. In this paper, we review the recent major accomplishments in bioinspired solution methods and tools for SCM systems. In particular, we concentrate on the modeling and optimization methods based on a class of metaheuristics inspired by biologically living beings, including genetic algorithms (GAs) [5], evolutionary programming (EP) [6], evolution strategies (ESs) [7, 8] and differential evolution (DE) [9], swarm intelligence [10, 11], and artificial immune [12]. These heuristic methods usually do not require deep mathematical knowledge, and have been demonstrated to be quite useful and efficient in optimization search for large-scale problems. We believe that this work will help researchers and practitioners to gain knowledge about the major developments emerged throughout the years and find valuable approaches that can be referred in the research or applied in the practice of SCM modeling and optimization.

The rest of the paper is synthesized as follows: Section 2 describes modeling and optimization methods based on GA, Section 3 depicts three other evolution-related methods including EP, ES, and DE, Section 4 describes methods based on swarm intelligence, and Section 5 introduces some other biological methods. Finally we discuss some future trends in Section 6 and conclude in Section 7.

## 2. Genetic Algorithms

### 2.1. Design of Supply Chain Network

Network design plays a key role in achieving efficient and effective management of SCM systems. Typically, a supply chain can be represented as a form of multistage-based structure, the optimal design of which has been recognized as *NP*-hard problems that combine the multiple choice knapsack problem with the capacitated location-allocation problem [13]. The first attempt to use the GA approach to solve the SCM network design problems has been proposed by Zhou et al. [14]. They developed a balanced star-spanning forest formulation for encoding the solutions and then used uniform crossover and exchange mutation operators in the algorithm. The experiment showed that for a maximum of 10 distributors and 100 customers, the algorithm can balanced all the distributors. Gen et al. [15] proposed a set of spanning tree-based for a class of network design problems such as degree-constrained minimum spanning tree problems, capacitated minimum spanning tree problems, fixed charge transportation problems, and network topological design problems, which were applied to some real-world SCM systems.

**Figure 1:**
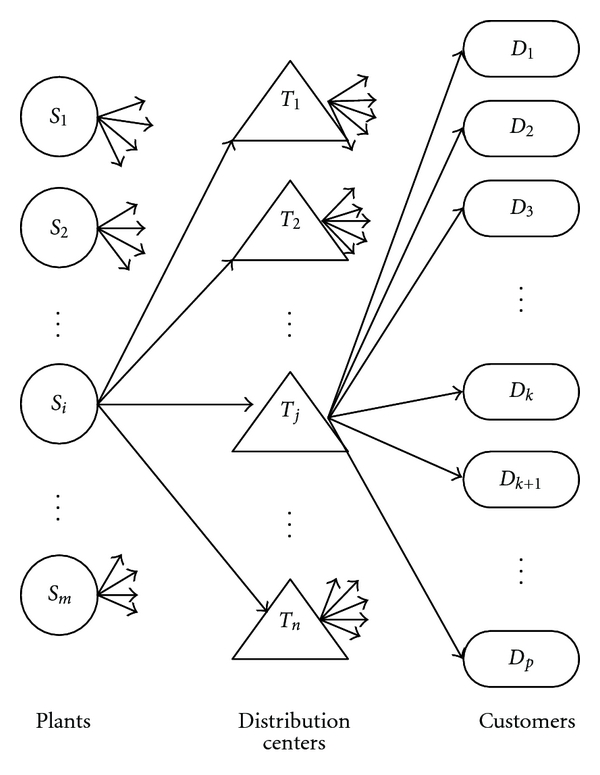
Illustration of the two-stage supply chain network design problems.

A multistage distribution problem is a standard one with supply chain network design. Many works focus on the two-stage supply chain distribution problem [16–20], which can be represented in Figure 1. That is, each of the *m* plants can ship to any of the *n* distribution centers, and each of the *n* distribution centers can ship to any of the *p* customers. Typically, each plant *i* has *s*_*i*_ units of supply, each customer *k* has *d*_*k*_ units of demand, and each distribution centre *j* has *t*_*j*_ units of stocking capacity, and the main purpose is to minimize the total cost which may include transportation cost, fixed cost, and transportation time.

Aiming at the demand allocation optimization of a two-stage and single product supply chain design problem, Chan et al. [18] implemented a multicriteria optimization algorithm which combines GA with the decision-making technique of the Analytic Hierarchy Process (AHP). Jawahar and Balaji [19] considered a two-stage distribution problem of a supply chain that is associated with a fixed charge and presented a GA that evolves the solution for best fitness of total cost of distribution. By fine-tuning its parameters, the algorithm can work out optimal solutions for small-size problem instances, but its performance on large-size instances has not been demonstrated. Feng and Zhang [20] extended the problem by involving multiple transportation modes and proposed a GA that can deal with middle-size problem instances.

**Figure 2:**
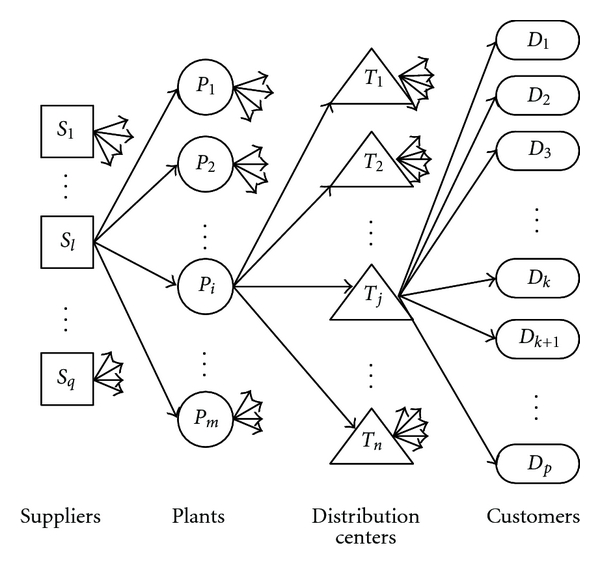
Design problem in three-stage supply chain networks.

 Since that today's market environment becomes more and more complex, supply chains with multiple (three or more) stages are common. To model a single product three-stage supply chain network (as illustrated in Figure 2), Altiparmak et al. [21] proposed a GA that uses a three-segment chromosome string which is decoded through a backward procedure from the third stage up to the first stage connecting suppliers and plants. They also extended their priority-based encoding in a new GA for the multi-product case in [22]. However, their decoding structure can require some repair procedures when the upper limits are exceeded; otherwise the capacities will be not enough to meet the customers' demand. Costa et al. [23] presented a new chromosome encoding and a complementary decoding procedure able to overcome the drawbacks and thus improve the efficiency and effectiveness of three-stage supply chains.

Considering that in a multistage supply chain network the flow can be only transferred between two consecutive stages, Yeh [24] presented a memetic algorithm for the design optimization of distribution network, which combined GA, linear programming, a greedy-heuristic based method, and three local search methods. Yun et al. [25] indicated that, when applying GA to multistage-based supply chains, conventional GA can do global search but there is no way for local search around the convergence area generate, and they proposed a new hybrid GA with an adaptive local search scheme which can automatically control whether local search technique is used in GA loop or not, and thus can reliably solve various optimization problems without trial-and-error experimentation.

### 2.2. Supply Chain Planning and Scheduling

The success of SCM highly depends on the timely and efficient production/distribution in the supply chain network, which can be typically regarded as a combination of planning and scheduling problems, each seriously affected by nearly prohibitive combinatorial complexity. In general, the development of an effective approach for SCM planning and scheduling can be divided into two steps:

(1) developing a precise mathematical model of the problem considered, explaining the objective function(s), decision variables, and problem constraints;

(2) selecting one or more effective metaheuristics suited to the problem, and developing a problem-solving algorithm-based on the heuristics.

Şerifoğlu and Ulusoy [26] considered the problem of scheduling independent jobs on identical parallel machines to minimize earliness/tardiness, which incorporates distinct due dates and arrival times for jobs, different processing rates for machines, and sequence-dependent set-up times into the problem formulation. They developed a GA with a new multicomponent uniform order-based crossover (MCUOX) operator and demonstrated that the GA outperforms other simple heuristic methods especially in larger-sized problems. Min and Cheng [27] also proposed a GA for solving the problem of scheduling identical parallel machines, where the objective is formulated as the minimization of the makespan.

Garcia et al. [28] considered the problem of scheduling a single production plant in order to satisfy delivery time constraints. They proposed two approaches, an exact method for simple cases and a GA for instances of more realistic size. Feng et al. [29] considered the problem of scheduling a single depot equipped with a fleet of vehicles with identical capacity and fixed loading/unloading times. They proposed a GA for searching a production sequence maximizing a predefined performance index, taking truck waiting times and a penalty for violating the unloading continuity of multitruck orders into consideration.

In [30], Lee et al. studied an operation-level advance planning and scheduling problem in supply chain, the model of which is to determine the best schedule using alternative operation sequences and machines, considering scheduling with outsourcing and strong constraints of the due dates of customer orders. They developed a genetic algorithm-based heuristic to solve it. Karabuk [31] studied the assignment of jobs to suppliers and aims to determine optimal production scheduling that minimizes the makespan, where each supplier requires a different length of time to process each job. The research proposed an adaptive GA with a new dominated gene crossover operator to solve this problem. Zegordi et al. [32] considered the scheduling of products and vehicles in a two-stage supply chain environment, assuming that the various output products occupy different percentages of each vehicle's capacity and proposed a gendered GA with two different chromosomes with nonequivalent structures that performs better than standard GA with a unique chromosomal structure.

There are also a number of reports on the applications of genetic algorithm in different trades, for example, [33–40]. Considering a semiconductor supply chain for reflecting the nonlinear throughput time of manufacturing, Chidambaram and Armbruster [33] proposed a hybrid LP and GA framework to solve the non-linear programming problem in order to avoid significant differences between the planned and realized output. Naso et al. [34] considered the problem of finding an optimized schedule for the just-in-time production and delivery of ready-mixed concrete on a set of distributed and coordinated production centers. They proposed a hybrid evolutionary algorithm in which the GA constitutes the core of the search strategy, while multiple heuristic rules called in specific circumstances contribute to reconstruct a feasible solution that satisfies all the constraints and objectives. The algorithm can guarantee the determination of a feasible schedule for any given set of requests and can address the highly complex scheduling problem of an entire supply-chain for just-in-time production. Dong and Ding [35] introduced a dynamic berth allocation model for container terminal and proposed a GA-based heuristic method that improves the existing research on static berth allocation models. The GA-based approach explores detailed capabilities of a complex problem solution using these two encoding methods. Recently, Delavar et al. [36] considered coordinated scheduling of production and air transportation and proposed two GA approaches to optimize customer service at minimum total cost: the first uses a portion of the search time to seek for the best transportation allocation and dedicates the remaining time to search for the best production-transportation solution; the seconds allocates some definite number of generations to search for the best production-transportation solution only after finding a better solution in each generation of transportation. In both algorithms, the Taguchi parameter design method was employed to adjust the parameters.

### 2.3. Multiobjective Optimization

In most cases, the design, planning, and scheduling of complex supply chains will involve trade-offs among different goals. Since 2000, multiobjective optimization of SCM systems has gained a lot of interests of the researchers. Generally, for a multiobjective optimization problem we need to search for a set of Pareto optimal solutions rather than a single optimal one. Using a minimization problem for illustration, let *f*_1_, *f*_2_,…, *f*_*m*_ be objective functions; a solution **x** is said to dominate **y** if and only if

(i) *f*_*i*_(**x**) ≤ *f*_*i*_(**y**)  for all  *i* ∈ {1, 2,…, *m*},
(ii) *f*_*i*_(**x**) < *f*_*i*_(**y**) for existing *i* ∈ {1,2,…, *m*}.

Regarding this, a Pareto-optimal front of the problem consists of all solutions for which the corresponding objective vectors cannot be improved in a given dimension without worsening another [41].

A typical multiobjective genetic optimization algorithm was proposed by Chan and Chung [42] for simultaneously minimizing the total cost of the system, total delivery days, and the equity of the capacity utilization ratio for manufacturers. In [43] Chan et al. proposed a hybrid GA for production and distribution problem, which utilized AHP to construct these criteria and calculate the fitness value of chromosome, and considered operating cost, service level, and resources utilization as objectives. Considering that all organizational units that participate on a single SC network are distributed by nature, constrained, and self-interested, Al-Mutawah et al. [44] used a distributed multiobjective GA to solve a three-subchain optimization problem, and their test results showed that the distributed GA provided an improved computational performance, because real-world supply chain applications are distributed in nature-distributed approach.

Altiparmak et al. [45] considered SCM optimization problem with three objectives: minimizing total cost, maximizing of customer services, and maximizing capacity utilization balance for distribution centers. To deal with the objectives and enable the decision maker for evaluating a number of alternative solutions, they proposed a GA which was designed to generate Pareto-optimal solutions considering two different weight approaches. Farahani and Elahipanah [46] adopted a hybrid non-dominated sorting GA to optimize total cost and service level for JIT distribution in a supply chain, whose results were compared with Lingo software to evaluate the performance of proposed algorithm. Che and Chiang [47] established a multiobjective optimization mathematical model for the build-to-order supply chain model which are defined as “the system that produces goods and services based on individual customer requirements in a timely and cost competitive manner by leveraging global outsourcing, the application of information technology and through the standardization of components and delayed product differentiation strategies” [48]. Considering three evaluation criteria including costs, delivery time, and quality, they proposed a modified Pareto GA to improve efficiency of the crossover and mutation operators of basic Pareto GA.

## 3. EP, ES, and DE

Besides GA, other evolutionary algorithmic methods have also been applied to many SCM modeling and optimization problems. EP was devised in order to evolve finite state machines for the prediction of events on the basis of former observations and has been demonstrated useful for searching the optimum of nonlinear functions [49]. Huang and Lu [50] proposed an interactive EP approach based on the relativistic error and selection of some other parameters to improve initial value determination, mutation, and variance parameter selective operation. The simulation results of supply chains showed that the improved EP is much more appropriate of nonline model with a great volume of data. Based on EP approach, Li et al. [51] proposed a heuristic strategic safety stock optimization algorithm for reverse logistics SCM considering the modeling complexity of external as well as internal product returns and reuses of supply chains.

Original ES uses a mutation operator that produces a single descendent from a given ancestor [7, 8], denominated ES-(1 + 1), and was progressively generalized to ES-(*μ* + *λ*), that is, several ancestors (*μ* > 1) and descendents (*λ* > 1) in each generation. Homberger [52] proposed an approach that combines the (1 + *λ*)-selection procedure with the Borda maximin voting rule, to coordinate decentral planning of a group of independent and self-interested decision makers, who are searching for an agreeable contract regarding multiple interdependent issues, in the case of asymmetric information presented. In [53] Dalkilic et al. developed an ES algorithm to solve a multiple-supplier multiple-item problem with stochastic lead times, which was successfully applied to some real-world healthcare SCM cases.

DE approach combines simple arithmetic operators with the classical operators of crossover, mutation, and selection to evolve a randomly generated starting population to a final solution. It is similar to a (*μ* + *λ*) ES, but in DE the mutation is not done via some separately defined probability density function [54]. Routroy and Kodali [55] developed a DE algorithm for minimizing the total systemwide cost, which consists of supply chain inventory capital, supply chain ordering/set-up cost, and supply chain stock-out cost. The result showed that the algorithm helps in determining ordering/production quantity and inventory/service level that should be maintained by each member of the supply chain. The algorithm was further extended for multiechelon supply chain inventory problems [56] and the problems with demand and leadtime uncertainty [57]. Prasertwattana and Shimizu also [58] applied a similar DE algorithm to optimize material ordering and inventory control of SCM systems.

DE was used for multiobjective optimization of SCM systems first by Babu and Gujarathi [59]. In their algorithm, crossover is carried out between the target vector and the noisy random vector to generate a trial vector, the cost of trial and target vectors is compared, and the variables corresponding to best cost are passed into next generation. The algorithm was successfully applied to a three-stage SCM problem. Dos Santos Coelho and Lopes [60] firstly developed a chaotic DE algorithm for the optimization of a supply chain, which was based on different DE approaches combined with chaotic sequences and led to better results than basic DE approaches. In [61] Xu et al. proposed a migration DE algorithm by imitating nomadic migration for this supply chain problem and presented an ensemble method based on different DE methods for not only avoiding the premature convergence but also improving the global search capability.

Falcone et al. [54] compared the performance of GA, EP, ES, and DE based on a case of integrated production-inventory-distribution SCM system. Their results showed that the robustness of the evolutionary methods is in general, and the efficiency of DE, in particular, suggests their great utility for the supply chain optimization problem.

## 4. Swarm Intelligence

### 4.1. Ant Colony Optimization

Ant colony optimization (ACO) algorithm mimics the behavior of real ants living in colonies that communicate with each other using pheromones in order to accomplish complex tasks such as establishing a shortest path from the nest to food sources [62]. Silva et al. [63] proposed an ACO algorithm for distributed optimization of a logistic system, but the work only considered only the allocation of suppliers in the system. In a successive work [64], the authors modeled a distributed optimization problem for a generic supply chain with suppliers, logistics, and distributers and developed an ACO algorithm that allows the exchange of information between different optimization problems by means of a pheromone matrix. The experimental results showed that the approach can significantly improve global supply chain performance with respect to other simple decentralized approaches.

Wang [65] studied the partner selection and production-distribution planning problem in a supply chain with the losses of production, which is called the defective supply chain. He developed for this problem a two-phase ACO algorithm, which finds out the combination of the maximum yield rate and the minimum number of partners in the first phase and implements the distribution with the partners and seeks out the minimum value of T-score in the second phase. Comparative numerical experiment showed that his algorithm achieves better performance than the common single-phase ACO algorithms.

In a very recent work, Moncayo-Martínez and Zhang [66] studied the multiobjective ACO for supply chain optimization. They considered the problem for minimizing the total cost while keeping the total lead-times within required delivery due dates. They formulated the design problem into an ACO optimization form and implemented a number of ant colonies in a sequence to explore the solution space and search for successively better nondominated set of supply chain designs.

### 4.2. Particle Swarm Optimization

Particle swarm optimization (PSO) [10] is another population-based global optimization technique that enables a number of individual solutions, called particles, to move through a hyperdimensional search space to search for the optimum. Each particle has a position vector and a velocity vector, which are adjusted at iterations by learning from a local best found by the particle itself and a current global best found by the whole swarm. Modeling a system where multiple-candidate solution coexists and collaborates simultaneously, PSO approaches embed problem-solving attempts in a social network and are suitable in nature for the optimization of very complex systems [67] and thus have been successfully applied in the research of SCM, for example, [68–74].

Izquierdo et al. [68] applied a PSO algorithm to a supply chain for searching optimal biomass flows from sources to energy production plants. Kadadevaramath et al. [70] proposed a PSO algorithm for the modeling and optimization of a four-stage supply chain and gained satisfying results. Bachlaus et al. [71] considered the design of a multiechelon supply chain network that integrates production, distribution and logistics activities and developed a hybrid PSO algorithm based on Taguchi robust design optimization tool [75]. The algorithm incorporates the characteristics of statistical design of experiments and random search techniques, which is an attractive way for determining flexible location and distribution strategies.

In [73] Sinha et al. considered the optimization of resource allocation for agents in a petroleum supply chain. They developed a coevolutionary PSO algorithm with two populations, and the decision vectors and the Lagrangian multipliers are taken to be constant in the first population and to be variables in the second population. The algorithm also uses a Cauchy random number distribution which is proved to be much better than a Gaussian distribution.

Soares [76] et al. utilized four kinds algorithms (including EP, GA, PSO, and EDA—estimation of distribution algorithm) to solve a multiple-retailer SCM problem, which is for finding an optimal balance of quantities ordered from suppliers and acceptable lead time costs while taking into account limiting factors such as the time each retailer will wait for a backorder. According to the results on the test-suite, three PSO algorithms of the 32 attempted algorithms demonstrate great flexibility and high performance.

### 4.3. Artificial Bee Colony Optimization

There are several optimization algorithms [77–80] that simulate the intelligent foraging behavior of a honeybee swarm. A more recent and popular approach is the artificial bee colony (ABC) algorithm that divides a bee colony into three groups, namely, employed bees exploiting on current food sources, onlookers waiting in the hive for choosing existing food sources, and scouts bees exploring new food sources. In [81] Kumar et al. analyzed the complexities of a remanufacturing problem in which the return rate is a function of environmental factor and proposed an ABC algorithm for the problem model. In their test results, the ABC algorithm significantly outperformed a PSO algorithm for comparison. In [82], Pal et al. considered a problem of integrated procurement, production, and shipment planning for a three-echelon supply chain. They developed two ABC-based algorithms for optimizing the order scheduling and production-shipment planning to achieve a minimum cost.

Considering the problem of a milk production, scheduling, and supply network design with extensively multiple conflicting objectives, Banerjee et al. [83] proposed a Pareto BCO approach, which was demonstrated to be better than some other bioinspired algorithms by simulation and comparison. For improving machine utilization and reducing cycle-time in manufacturing industry, Li et al. [84] applied a Pareto bee colony optimization (BCO) algorithm for a multiobjective flexible job shop scheduling problem and gained good computational result.

## 5. Other Methods from Biological Intelligence

Provoked by the theoretical immunology, observed immune functions, principles and models, artificial immune system (AIS) stimulates the adaptive immune system of a living creature to unravel the various complexities in real-world engineering optimization problems [85]. Shukla et al. [86] employed an AIS approach to a batch sequencing problem in a multistage supply chain, which considers three objectives including minimization of lead time, blocking time, and due date violation, and the experimental results showed that the AIS outperforms GA and simulated annealing (SA).

Prakash and Deshmukh [85] considered a multiple vendor transportation problem with time and cost criteria and proposed an AIS algorithm strengthened by a fuzzy logic controller (FLC) to solve the multicriteria problem. AIS works as an evolutionary search algorithm to find out the Pareto optimal front, whereas FLC is implemented to change the hypermutation rate adaptively on the basis of the fitness values at each iteration. They also employed a web-based supply chain to facilitate the SCM enterprise by e-learning.

In [87] Hajiaghaei-Keshteli considered a two-stage supply chain network of distribution centers and customers. To solve the problem for selecting some potential places as distribution centers in order to supply demands of all customers with minimum opening cost plus shipping cost, he, respectively, developed a GA and an AIS algorithm, and the results showed that the AIS algorithm exhibits robust performance improvements in large size problems versus GA.

## 6. Discussion

We have summarized the main bioinspired methods for SCM system design and optimization. It is deserved to note that swarm-based methods and artificial immune systems are not yet mature and thus are expected to gain more research interests. With the increasing importance and complexity of SCM systems, researchers are facing the challenges to promote the performance, reliability, and scalability of SCM problem-solving methods, and here we highlight the following future trends in bioinspired computation in SCM systems.

### 6.1. Hybrid

Different bioinspired methods have different design principles and application areas. As we mentioned in previous sections, there are a number of studies that exploit the strengths of several individual methods to obtain a more powerful approach to dealing with complex SCM problems, and to a great extent, these hybrids methods are shown to be more competitive than individual methods. It can be anticipated that future research will continuously put great emphasis on the hybridization of bioinspired methods, for example, swarm-based evolutionary algorithms [88–90], and the hybridization of bioinspired methods with other approaches such as local search [91], tabu search [92], and simulated annealing [93].

### 6.2. Extension with New Computing Paradigms

We are seeing that innovative informational/computational paradigms, such as chaotic systems, quantum informatics, and DNA computing, provide valuable inspiration to create new heuristics for complex optimization problems including a host of *NP*-hard problems. Thus, the extensions of current bioinspired methods based on these new paradigms are expected to achieve dramatic improvement on computational performance. For example, chaotic sequencing and local search operations have been successfully applied for helping evolutionary algorithms avoiding premature convergence effectively [94, 95]. Also, quantum-inspired evolutionary algorithms are regarded as one of the three main research areas related to the complex interaction between quantum computing and evolutionary algorithms [96–98] and have been applied in some SCM optimization problems in very recent research [99]. These approaches are expected to show great promises for the future.

## 7. Conclusion

Today's SCM systems have to deal with ever-changing markets and intrinsic structural complexity emerging from virtually infinite number of interacting entities. Therefore, the community requires effective artificial intelligence methods and tools for modeling and optimizing large-scale complex supply chains. The paper has reviewed the recent development of bioinspired methods in SCM applications. Typical illustrations are addressed for evolutionary algorithms including GA, EP, ES, and DE, swarm-based intelligent algorithms including ant colony, particle swarm and artificial bee colony, and other bioinspired methods like AIS. Representative works are summarized for helping readers to have a general overview of the state of the art and to easily refer to suitable methods in practical solutions. Over the last decade, bioinspired methods have experienced a rapid growth and have successfully applied to the design and optimization of highly complex systems such as SCM systems. The fruits of these researches are continuously becoming new technological solutions to new open problems, and the full potential is far from being reached.
